# High-Frequency Electrical Modulation of the Superior Ovarian Nerve as a Treatment of Polycystic Ovary Syndrome in the Rat

**DOI:** 10.3389/fphys.2018.00459

**Published:** 2018-05-01

**Authors:** Victor Pikov, Arun Sridhar, Hernan E. Lara

**Affiliations:** ^1^Huntington Medical Research Institutes, Pasadena, CA, United States; ^2^Galvani Bioelectronics, Stevenage, United Kingdom; ^3^Centre for Neurobiochemical Studies in Endocrine Diseases, Faculty of Chemistry and Pharmaceutical Sciences, University of Chile, Santiago, Chile

**Keywords:** superior ovarian nerve, kilohertz frequency alternating current, high-frequency stimulation, high-frequency block, polycystic ovary syndrome

## Abstract

The polycystic ovary syndrome (PCOS) is the most prevalent ovarian pathology in women, with excessive sympathetic activity in the superior ovarian nerve (SON) playing an important role in inducing the PCOS symptoms in the rats and humans. Our previous studies have shown that surgical transection of the SON can reverse the disease progression, prompting us to explore the effect of the kilohertz frequency alternating current (KHFAC) modulation as a method of reversible non-surgical suppression of the nerve activity in the rodent model of PCOS. 56 animals were randomly allocated to three groups: the Control group (*n* = 18), the PCOS group (*n* = 15), and the PCOS + KHFAC group (*n* = 23). The physiological, anatomical, and biochemical parameters of ovarian function were evaluated during the progression of the experimentally-induced PCOS and during long-term KHFAC modulation applied for 2–3 weeks. The KHFAC modulation has been able to reverse the pathological changes in assessed PCOS parameters, namely the irregular or absent estrous cycling, formation of ovarian cysts, reduction in the number of corpora lutea, and ovarian norepinephrine concentration. The fertility capacity was similar in the PCOS and the PCOS + KHFAC groups, indicating the safety of KHFAC modulation approach. In summary, these results suggest that the KHFAC modulation approach of suppressing the SON activity could become a useful treatment modality for PCOS and potentially other pathological ovarian conditions.

## Introduction

Polycystic ovary syndrome (PCOS) is the most common ovarian pathology during reproductive age; it is characterized by hyperandrogenism and the polycystic ovary phenotype (Azziz et al., 2009). PCOS affects up to 17% of women and is often associated by endocrine/metabolic disorders (March et al., 2009). One of the principal symptoms of PCOS is anovulation, resulting in irregular menstruation and infertility. In addition, androgen secretion by the cysts in PCOS patients leads to masculinizing effects, such as acne and hirsutism. Recently, suppression of sympathetic activity by acupuncture was found to reduce the PCOS symptoms (Stener-Victorin et al., 2008). Current treatments of PCOS, such as estrogen receptor modulators, gonadotropins, laparoscopic ovarian drilling, weight loss and exercise, focus on the symptoms rather than the underlying cause and are moderately effective. For example, estrogen receptor modulators (e.g., clomiphene and metformin) and gonadatropins target the hormonal symptoms to induce regular cycling and ovulation, while laparoscopic ovarian drilling stimulates ovulation by decreasing the mass of tissue, the success of the procedure is in direct relation to the capacity to decrease androgen production in patients (Amer et al., 2004). Present PCOS therapies do not account for complexity of the brain–ovary connection in the human body. Indeed, while ovulation is controlled by the pituitary-secreted gonadotropins (luteinizing hormone and follicle-stimulating hormone), it is also strongly influenced by the sympathetic neural control (Lara et al., 2002). Mammalian ovary receives a dense innervation of post-ganglionic sympathetic nerves, originating from the ovarian ganglion and from the celiac and renal plexi (Burden et al., 1985; Curry et al., 1985). The ovary receives its sympathetic innervation via two nerves: (1) the ovarian plexus nerve, which is associated with the ovarian branch of the uterine artery (Neilson et al., 1970), and (2) the superior ovarian nerve (SON), which is at least partially associated with the ligament of ovary and also known as the suspensory ligament or the infundibulopelvic ligament (Neilson et al., 1970; Lawrence and Burden, 1980). The SON fibers predominantly innervate the secretory components of the ovary, i.e., interstitial glands and follicles, whereas the ovarian plexus nerve fibers are mostly perivascular (Lawrence and Burden, 1980). Although there is some variation in the way that sympathetic nerves reach the ovary, no differences have been found in the intraovarian distribution of sympathetic fibers, which is similar in all mammalian species (although the density of the network varies considerably among them, Jacobowitz and Wallach, 1967). Norepinephrine is the main neurotransmitter present in the ovary (Lara et al., 1990, 2002; Greiner et al., 2005). The innervation of the gland has been shown to be involved in the regulation of ovary specific functions, such as steroidogenesis and early follicular development (Lara et al., 2002; Greiner et al., 2005) by activating B-adrenergic receptors present in cells of the ovarian follicle.

Transection of the SON, which carries the bulk of the sympathetic innervation to ovarian endocrine cells has been observed to restore estrous cyclicity and ovulation (Barria et al., 1993). In contrast, a sustained increase in sympathetic activity by estradiol administration (Lara et al., 2000), chronic sympathetic stress (Dorfman et al., 2003), or pharmacological β-adrenergic receptor activation (Luna et al., 2012) causes the appearance of a polycystic phenotype in the rat ovary, which in many aspects resembles the PCOS in women.

In this study, the effect of suppressing the SON activity with the kilohertz frequency alternating current (KHFAC) was evaluated as a potential treatment for PCOS. KHFAC has been previously shown to produce an effective and reversible block of nerve conduction in somatic and autonomic nerves (Kilgore and Bhadra, 2004, 2014; Williamson and Andrews, 2005; Patel and Butera, 2015; Patel et al., 2017).

## Materials and Methods

### Study Design

All animal studies were carried out in accordance with the animal protocols approved by the Huntington Medical Research Institutes (HMRI) Institutional Animal Care and Use Committee and the Bioethics Committee of the Faculty of Chemistry and Pharmaceutical Sciences at the Universidad de Chile (UCh). They also complied with national guidelines (CONICYT Guide for the Care and Use of Laboratory Animals) and in accordance with GlaxoSmithKline Policy on the Care, Welfare and Treatment of Animals. Unless noted otherwise, the same procedures and animal protocols were used at the HMRI and UCh locations. Female Sprague-Dawley rats were randomly assigned to the following three groups:

(1) Control group: sham subcutaneous injection of 1 ml/kg sesame oil, no sham surgery;
(2) PCOS group: PCOS was induced as previously described (Cruz et al., 2012). PCOS was induced at 4 weeks of age by a single subcutaneous injection of Estradiol valerate (EV, B-estradiol-17-valerate, Sigma) at supra-physiological dose of 10 mg/kg, dissolved in 1 ml/kg sesame oil; at 7 weeks post-injection (i.e., 11 weeks of age), the animal was anesthetized and the surgery was performed to remove the right ovary. In two animals (both at HMRI location), the cuff electrode was implanted on the left SON during the surgery for removing the right ovary;
(3) PCOS + KHFAC group (*n* = 23): PCOS was induced by EV at 4 week of age (same as in the PCOS group). At 7 weeks post-injection, the animal was anesthetized and the surgery was performed to remove the right ovary and to implant the cuff electrode on the left SON; starting at 8 weeks post-injection, the KHFAC modulation was applied to the SON for 2–3 weeks.

Fifty-six animals were used in this study. These animals were randomly allocated to three groups: the Control group (*n* = 18, including *n* = 13 at HMRI and *n* = 5 at UCh), the PCOS group (*n* = 15, including *n* = 2 at HMRI and *n* = 13 at UCh), and the PCOS + KHFAC group (*n* = 23, including *n* = 3 at HMRI and *n* = 20 at UCh). In the PCOS and PCOS + KHFAC groups, the right ovary was removed and PCOS was induced at the age of 28 ± 3 days by a single subcutaneous injection of EV. In the PCOS + KHFAC groups, at the time of EV injection and ovary removal, the cuff electrode was chronically implanted on the left SON. At HMRI location, KHFAC modulation was applied at 77 ± 3 days of age for 21 ± 3 consecutive days (*n* = 3). In comparison, at UCh location, KHFAC modulation was applied at 88 ± 2 days of age (*n* = 20) and due to the animal concern (excessive stress levels noted in the animals), the KHFAC modulation was discontinued a week earlier, at 15 ± 4 days. The stress related symptoms (e.g., excessive level of activity) were observed visually during daily animal inspection at the vivarium. As soon as these symptoms were detected, KHFAC modulation was discontinued in all animals, and the stress symptoms disappeared within 2–3 days. For that reason, the estrous cycling data from 12 animals at UCh was excluded from the analysis. The test for histology, NE concentration, and fertility capacity were performed at least 7 days after stopping the KHFAC modulation, to minimize the earlier acute stress response experienced by the animals and not to have a significant effect of these parameters. Notwithstanding some variability in the duration of KHFAC modulation, all animals in the PCOS + KHFAC group were analyzed together.

Four primary outcome measures were evaluated in the animals:

• Estrous cycle phase, evaluated daily by vaginal cytology;
• Fertility capacity, evaluated at 12^th^ week after EV injection (UCh location only);
• Ovarian morphological phenotype, evaluated at 100 ± 10 days of age;
• Ovarian norepinephrine (NE) concentration, evaluated at 100 ± 10 days of age (UCh location only for the Control group *n* = 5 and for the PCOS group *n* = 13, UCh and HMRI locations for the PCOS + KHFAC group: *n* = 8 at UCh and *n* = 2 at HMRI shipped to UCh for processing).

### Surgical Procedures for the Ovary Removal and Cuff Electrode Implantation on the SON

Animals in the PCOS and the PCOS + KHFAC groups were anesthetized for the removal of right ovary. In addition, the cuff electrodes have been implanted on the SON in the PCOS + KHFAC group (and two animals at the PCOS group at HMRI location). General anesthesia was induced and maintained with Ketamine/Xylazine (90/9 mg/kg, IP). The scalp and lateral skin at mid-lumbar level (over the ovaries) were shaved and animal was placed on a heated pad to maintain body temperature. First longitudinal off-midline skin incision was made at a mid-lumbar level on the right side. The underlying muscles were dissected, and the skin/muscles were retracted. The peritoneal incision was then made to access the peritoneal cavity. The adipose tissue was pulled away until the ovary surrounded by a variable amount of fat was identified. The ovary was ligated, removed, and saved for measuring the NE concentration. The peritoneal and muscle incisions were sutured with 5-0 PDS absorbable suture using a single continuous suture pattern. The skin incision was then sutured using the continuous subcutaneous suturing technique with 5-0 monofilament nylon followed by an application of veterinary cyano-acrylate glue to prevent the animal from removing the suture. In the animals from the PCOS + KHFAC group and the PCOS group at HMRI location, second longitudinal off-midline skin incision was made at the contralateral (left) side. The skin/muscles were similarly retracted and the superior ovarian ligament with embedded SON nerve was exposed. The silicone-based split-cylinder bipolar cuff with 0.5 mm inner diameter, 1.5 mm outer diameter, 8 mm length, and two outer flaps (item 1041.2154.01, Cortec GmbH) was placed on the ligament. One or two 7-0 prolene sutures were placed around the cuff to secure it on the ligament. The cuff contained two longitudinally spaced electrodes (center-to-center distance of 4 mm) made from platinum foil sandwiched between two layers of silicone, with each electrode consisting of two laser-exposed 50-μm-recessed circumferentially spaced contacts (edge-to-edge distance of 0.3 mm), connected by non-exposed stretchable meandering metal tracks (Ordonez et al., 2014). Both contacts had the circumferential length of 0.7 mm, width of 0.65 mm, resulting in the exposed electrode area of 0.91 mm^2^. Two wires exited from the cuff longitudinally, they were made from 70-μm-diameter MP35N/Silver DFT^®^ conductor coated with polyesterimide (Fort Wayne Metals). At UCh location, the cuff wires were soldered to a 2-pin percutaneous connector (801-87-002-10-001101, Preci-Dip). At HMRI location, the cuff wires plus the platinum ground electrode were soldered to a 6-pin percutaneous connector (MS363, Plastics One), with the ground electrode placed subcutaneously for electrochemical impedance (EIS) recordings. Finally, the longitudinal midline scalp incision was performed. The connective tissue and muscles were scraped from the skull. The wires and head-mounted percutaneous connector were tunneled subcutaneously (through a plastic guiding tube) and exteriorized at the scalp incision. The connector was affixed to the skull with the veterinary cyano-acrylate glue. Three or four self-tapping screws (#00-90, Stainless Steel 303, Fillister Head, 0.0625′′ length) were placed around the connector and the bone cement was applied. After the cement hardening, any potentially abrasive irregularities were smoothed to avoid skin irritation. The scalp was drawn up around the connector and sutured with 4-0 monofilament nylon. Closing of the peritoneal, muscle, and skin incisions over the SON cuff was performed as for the first incision. The animal was given Ketoprofen (2 mg/kg SC) prior to recovery from surgery. Children’s Ibuprofen (0.2 mg/ml final concentration) was added to drinking water for 5 days after surgery, and the antibiotic Septra (trimethoprim-sulfamethoxazole, 30 mg/kg, 0.1 mg/ml) was added to drinking water for 10 days after surgery.

### Electrochemical Impedance (EIS) Assessment

Electrochemical impedance measurements were obtained from the implanted cuffs at weekly intervals post-implantation. Restraint was provided by placing the animal in a conical plastic sleeve (Decapicone, Braintree Scientific, Inc.) with a small nasal opening to allow respiration and then closing the sleeve with staples during the 2-min testing period. The sinusoidal AC potential of ±10 mV was applied at the frequencies from 0.1 to 100 kHz using the Autolab potentiostat system (PGSTAT128N with FRA32M module, Metrohm) in a three-electrode configuration, with the working and counter lines connected to the cuff electrodes and the reference line connected to the platinum ground.

### KHFAC Modulation of the SON

The bipolar cuff was connected (through the head-mounted percutaneous connector) to the custom-made external wearable stimulator (StimAdjuster, Draper Labs) with an attached 600 mAh Lithium-Ion battery. The stimulator and battery were connecter to each other and to the head-mounted percutaneous connector and then placed inside a pocket in the rodent jacket, which was custom-made from a tear-resistant material (Super K Kote Ripstop) with Velcro straps. The jacket was mounted on the animal’s back and was replaced weekly with a clean one. The KHFAC stimulation was applied to the cuff electrode as the biphasic sinusoidal waveform with the current amplitude of ±1.5 mA and the frequency of 50 kHz. The stimulation was applied continuously for 2–3 weeks, with the batteries being replaced daily to avoid their depletion.

### Estrous Cycle Phase Assessment

The cycling phase was evaluated daily by vaginal cytology. Vaginal secretion was collected with a plastic pipette filled with 10 μl of normal saline (NaCl 0.9%) by shallow insertion of the pipette tip into the distal opening of vaginal cavity, while gently restraining the rat. Vaginal fluid was placed on the glass slide and observed under a light microscope. The proportion of epithelial cells (large, round, nucleated), cornified cells (irregular, without nucleus), and leukocytes (small, round) were calculated to define Proestrus (P) the day of ovulation; Estrus (E) the day after ovulation and Diestrus (D) the two previous days before P, as previously described (Marcondes et al., 2002).

### Fertility Capacity Assessment

Fertility capacity was evaluated at 12^th^ week after EV injection by performing the assisted mating (Urra et al., 2016). Assisted mating is performed by placing the male rat of proven fertility together with a female rat for 8 days (two full cycles). The female is added to the male’s cage to prevent territorial fighting. The mating success was confirmed by the presence of vaginal semen plug. The animal fertility was then determined at 7 days after a successful mating by (a) cessation of estrus cycle and (b) presence of embryonic vesicles in the dissected uterine horns and uterine cavity. Based on UCh experience in the evaluation of fertility capacity and determination of ovarian NE, these procedures were conducted at UCh only.

### Assessment of Ovarian Morphological Phenotype and Ovarian NE Concentration

Euthanasia was performed at 100 ± 10 days of age in the Control and the PCOS groups and the end of the KHFAC period in the PCOS + KHFAC group. The anesthesia was induced with Ketamine/Xylazine (90/9 mg/kg, IP), the ovary and SON were dissected, then the euthanasia solution (Pentobarbitol 200 mg/kg, IP) was injected into the heart. The ovary was cut in half, with one half post-fixed in 4% paraformaldehyde and used for histological evaluation, while the other unfixed half used for evaluation of NE concentration. The fixed half of the ovary was embedded in paraffin, cut at 10-μm sections using the microtome, mounted on the glass slide, and stained with H&E to evaluate the chronic ovary phenotype by quantifying the number of corpora lutea and cysts. The unfixed half of the ovary was homogenized with 0.4N perchoric acid, lyophilized, and the NE concentration was assessed with high-performance liquid chromatography (HPLC).

### Statistical Analysis

For statistical analyses data was evaluated using Minitab (v 17, Minitab, Inc.). The significance of the differences among three experimental groups was calculated by one-way ANOVA with Dunnett multiple comparison *post hoc* test. For within-animal comparison, the two-tailed *t*-test was used. Differences were considered significant at *P* < 0.05 and highly significant at *P* < 0.01.

## Results

### Electrochemical Impedance Assessment of Long-Term Reliability

**FIGURE 1:**
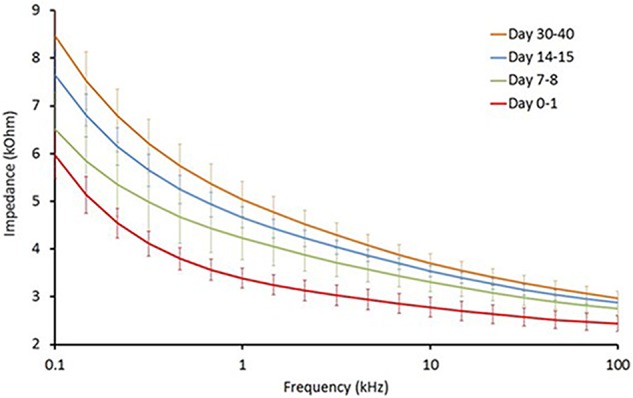
Electrochemical impedance (EIS) assessment of long-term reliability of the cuff contact with the SON (*n* = 5).

Long-term reliability of the cuff contact with the SON was assessed by the EIS in five animals from the PCOS and the PCOS + KHFAC groups at weekly intervals (at HMRI location). As expected, the cuff impedance gradually increased during first 30–40 days post-implantation, likely due to formation of a fibrous tissue sheath around the cuff (**Figure 1**). The impedance values, measured at 50 kHz, remained below 6 kOhm at all chronic timepoints, indicating that the applied voltage was below ±9 V (±1.5 mA ^∗^ 6 kOhm) and assuring us that the applied pulses were within ±10 V compliance of the stimulator.

### Estrous Cycling Activity

**FIGURE 2:**
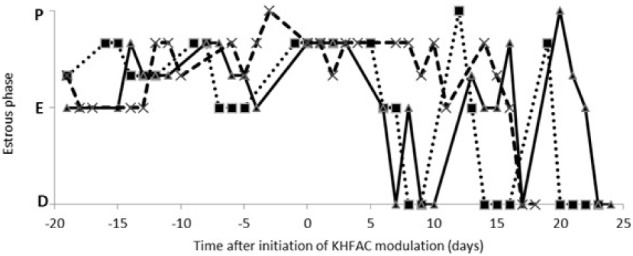
Estrous cycle dynamics in the PCOS + KHFAC group. Cycling activity has ceased prior to initiation of KHFAC modulation. Letters on the vertical axis indicate the estrous phases: proestrus (P), estrus (E), and diestrus (D). The traces represent three individual animals.

**FIGURE 3:**
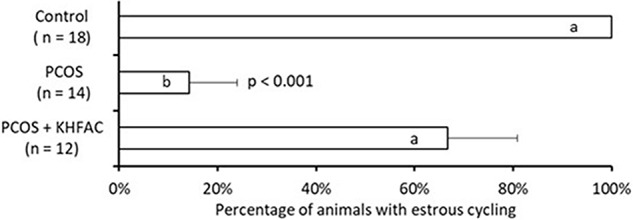
Percentage of animals in three experimental groups with estrous cycling. Results correspond to mean value + SEM of the number of animals shown in parenthesis. Different letters represent significant differences (*P* < 0.01) between the treatment groups.

The estrous cycle dynamics was evaluated in both the PCOS and the PCOS + KHFAC groups. In three PCOS + KHFAC animals at HMRI that were subjected to a 3-week-long KHFAC modulation, normal estrous cycling was restored at 7–17 days after the initiation of KHFAC. To avoid the challenges in visualizing the cycling dynamics for different KHFAC durations at HMRI (21 ± 3 days) vs. UCh (15 ± 4 days), **Figure 2** shows the cycling dynamics only for the PCOS + KHFAC animals at HMRI. **Figure 3** shows the percentage of animals in three experimental groups with estrous cycling: for the PCOS group, this indicates the persistent loss of cycling; and for the PCOS + KHFAC group, this indicates the recovery of cycling during the second and third week of KHFAC modulation.

The PCOS group exhibited lower percentage of animals with the estrous cycling, as compared to the control and the PCOS + KHFAC groups (**Figure 3**). Decrease in the PCOS group was highly significant (*P* < 0.001, ANOVA), while no decrease was observed in the PCOS + KHFAC group, indicating that the KHFAC modulation reversed the EV-induced suppression of estrous cycling. As mentioned in Section “Materials and Methods,” the PCOS + KHFAC animals that exhibited excessive stress levels were excluded from the analysis.

### Cyst and Corpora Lutea Analysis

**FIGURE 4:**
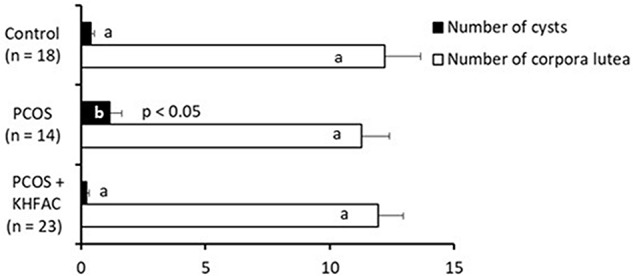
Between-animal comparison of ovarian morphological phenotype in three experimental groups. Results correspond to mean value + SEM of the number of animals shown in parenthesis. Different letters represent significant differences (*P* < 0.05) between the treatment groups.

Quantification of the ovarian morphological phenotype indicated the unchanged number of corpora lutea among the three groups and an increased number of cysts in the PCOS group, compared to the Control and the PCOS + KHFAC modulation groups (**Figure 4**).

**FIGURE 5:**
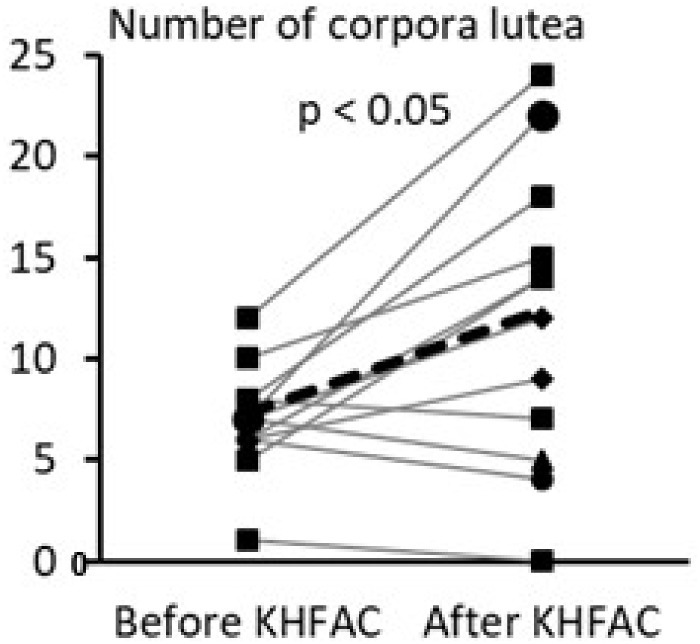
Within-animal comparison of ovarian morphological phenotype in 12 animals from the PCOS + KHFAC group. Individual values are shown as gray lines, while the group average is shown as the black line.

The within-animal comparison of the ovarian morphological phenotype was performed for the PCOS + KHFAC group between the right ovary removed at 7 weeks after EV injection (during the SON cuff implantation surgery and the left ovary dissected at 11–12 weeks after EV injection after completion of the KHFAC modulation period; **Figure 5**). 12 out of 23 animals in the PCOS + KHFAC group were evaluated in this Figure, since the right ovary was collected and processed for histology only in these animals (the left ovary was collected and processed for histology in all 23 animals, as shown in **Figure 4**). The within-animal comparison (before vs. after KHFAC) demonstrated a significant increase in the number of corpora lutea after the completion of KHFAC modulation.

### Ovarian Norepinephrine Concentration

**FIGURE 6:**
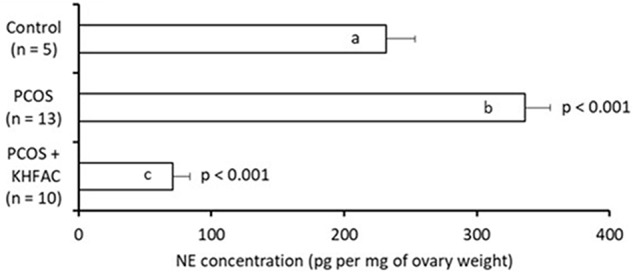
Evaluation of NE concentration in the ovary (normalized by the ovary weight) in three experimental groups. Results correspond to mean value + SEM of the number of animals shown in parenthesis. Different letters represent significant differences (*P* < 0.001) between the treatment groups.

The ovarian NE concentration was assessed in all three groups: the Control group – 5 animals (all at UCh), the PCOS group – 13 animals (all at UCh) and the PCOS + KHFAC group – 10 animals (8 animals at UCh, as the remaining 12 animals at UCh were used for fertility capacity assessment, and 2 animals at HMRI shipped to UCh for processing). The highest NE level was seen in the PCOS group (336 ± 19 pg/mg ovary), followed by the Control group (232 ± 22 pg/mg ovary) and the PCOS + KHFAC group (71 ± 13 pg/mg ovary) (**Figure 6**). The *post hoc* differences from the Control group were highly significant (*P* < 0.001), indicating considerable elevation of the NE release after the initiation of PCOS by EV and considerable suppression of the NE release following the KHFAC modulation.

### Fertility Capacity

**FIGURE 7:**
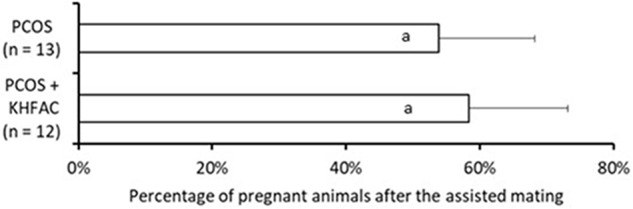
Evaluation of the fertility capacity, measured as the percentage of pregnant animals after the assisted mating, in the PCOS and the PCOS + KHFAC groups. Results correspond to mean value + SEM of the number of animals shown in parenthesis. Different letters represent significant differences (*P* < 0.05) between the treatment groups.

The fertility capacity in the Control group was not assessed in this study; instead, we used the historical data (Piquer et al., 2017) of 83 ± 3% pregnancy rate observed in 20 primiparous animals after the assisted mating, performed using the same procedure as in this study. The fertility capacity was assessed in 13 animals in the PCOS group and 12 animals from the PCOS + KHFAC group (all animals are from UCh location). Application of chronic KHFAC modulation in the PCOS + KHFAC group did not produce any detrimental effects on the fertility, as the percentage of pregnant animals was not significantly different in the PCOS group (54 ± 14%) and the PCOS + KHFAC group (58 ± 15%) (**Figure 7**).

## Discussion

This study evaluated several physiological, anatomical, and biochemical parameters of ovarian function after the experimentally-induced PCOS and long-term KHFAC modulation of the SON in the rodents. The KHFAC modulation has been able to reverse the pathological changes in these PCOS parameters, namely the irregular or absent estrous cycling, formation of ovarian cysts, reduction in the number of corpora lutea, ovarian NE concentration, and the fertility capacity. One limitation of this study is the lack of the PCOS + sham KHFAC group, where the animals are subjected to the same surgical implantation procedures as the animals in the PCOS + KHFAC group, but no KHFAC modulation is delivered through the implanted cuff electrodes. While having such a group would have been helpful, we believe that the safety aspect of cuff implantation is sufficiently addressed in the PCOS + KHFAC group by evaluating the physiological (**Figure 2**) and anatomical (**Figure 5**) parameters of ovarian function before vs. after the KHFAC modulation. Second limitation of the study is that KHFAC modulation was terminated after 2 weeks at UCh due to the appearance of stress-related symptoms (e.g., excessive level of activity), as compared to the 3 weeks of KHFAC modulation at HMRI. Once the KHFAC modulation was discontinued at UCh, the stress symptoms disappeared within 2–3 days. To remove any possible confounding effect of the stress response, we excluded 12 animals with elevated stress symptoms from the analysis of estrous cycling (**Figure 3**). These animals were however included in the evaluation of histology (**Figures 4**, **5**), NE concentration (**Figure 6**), and fertility capacity (**Figure 7**), as these tests were performed at least 7 days after stopping the KHFAC modulation, so the earlier transient stress response experienced by the animals was unlikely to significantly affect these ovarian parameters.

The mammalian ovary is involved in mediating two functions: (a) production of sex steroids and (b) maturation of the oocyte to be fertilized (ovulation). The EV model of PCOS produces early puberty (evidenced as earlier vaginal opening), anovulatory condition during adulthood (evidenced as either lacking transition from proestrus to estrus or complete anovulation), low levels of corpora lutea and appearance of follicular cyst (Lara et al., 1993; Dorfman et al., 2003; Bernuci et al., 2008; Cruz et al., 2012). Surgical transection of the SON recovers the cyclic ovulatory activity, and leads to higher number of corpora lutea and disappearance of cyst (Lara et al., 2000; Dorfman et al., 2003; Bernuci et al., 2008). In this study, similar recovery in the cyclic ovulatory activity, higher number of corpora lutea, and disappearance of cyst have also observed in the PCOS + KHAFC group. The restoration of normal estrous cycling became evident after 7–17 days of KHFAC duration, which corresponds to 2–4 estrous cycles in this species.

We speculate that the SON activity directly influences the ovarian function through the efferent innervation, although it also is possible that it may also affect the ovarian function indirectly by activating the afferent fibers to modulate the release of circulating hormones from the hypothalamus and pituitary (e.g., gonadotropin), which then influence the ovary by changing the blood flow in the ovarian artery (Kagitani et al., 2008). The KHFAC suppression of SON activity does not exclude either of these potential mechanisms, as the activity of both the afferent and efferent fibers could have been suppressed. However, the decrease in ovarian NE release after KHAFC supports the role of high NE concentration in the induction of the PCOS phenotype observed in rats and confirms the results of earlier studies from Dr. Lara’s group, demonstrating increased ovarian NE concentration in the PCOS phenotype induced by EV or by chronic-stress exposure (Lara et al., 1993; Dorfman et al., 2003). This study did not explicitly evaluate the completeness of SON activity suppression, however, the 79% reduction in the ovarian NE concentration indicates a rather significant degree of suppression of the SON activity, at least of its efferent fibers.

Finally, chronic application of the KHFAC modulation has been shown to be safe, as evidenced by similar fertility capacity in the animals previously subjected to KHFAC, as compared to the PCOS animals not subjected to the chronic nerve stimulation. In summary, the presented results suggest that the KHFAC modulation of the SON could be used for bioelectronic treatment of PCOS and potentially other pathological ovarian conditions. Such bioelectronic medicines could be tailored for each individual PCOS patient by applying a precise detection and modulation of the nerve signaling in a time- and dose-dependent manner (Birmingham et al., 2014).

## Author Contributions

VP and HEL equally contributed to the study design, data collection, analysis, and manuscript preparation. AS conceived the idea and participated in the study design, data analysis, and manuscript preparation.

## Conflict of Interest Statement

VP and AS are employees of Galvani Bioelectronics, a for-profit company. The other author declares that the research was conducted in the absence of any commercial or financial relationships that could be construed as a potential conflict of interest.
